# Supplementary material for: Causal Relationship between Aging and Anorexia Nervosa: A White-Matter-Microstructure-Mediated Mendelian Randomization Analysis
Source: Biomedicines. 2024 Aug 16;12(8):1874. doi: 10.3390/biomedicines12081874 (PMC11351342; doi:10.3390/biomedicines12081874)
Supplement: Supplementary file 1 [file biomedicines-12-01874-s001.zip › Supplementary Figure/Figure S4/Singe SNP plot of MR analysis for TL to ubm-b-1787.pdf]

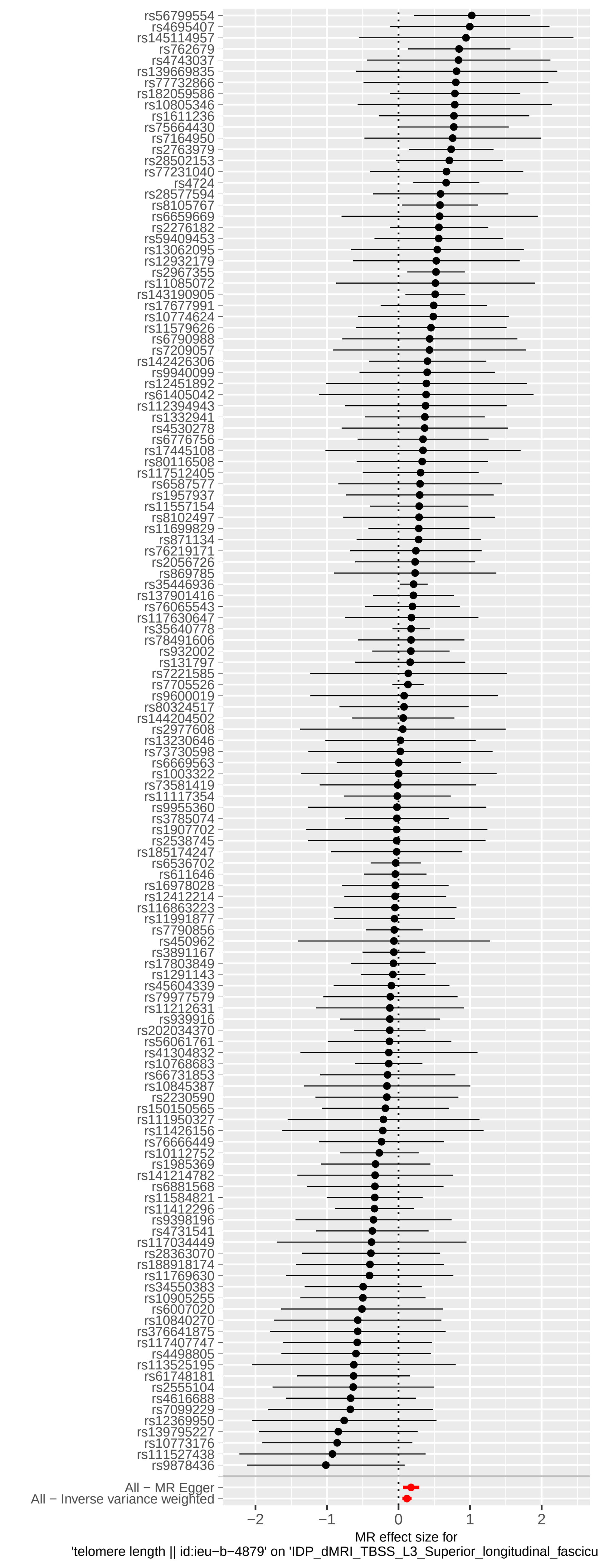
rs56799554
rs4695407
rs145114957
rs762679
rs4743037
rs139669835
rs77732866
rs182059586
rs10805346
rs1611236
rs75664430
rs7164950
rs2763979
rs28502153
rs77231040
rs4724
rs28577594
rs8105767
rs6659669
rs2276182
rs59409453
rs13062095
rs12932179
rs2967355
rs11085072
rs143190905
rs17677991
rs10774624
rs11579626
rs6790988
rs7209057
rs142426306
rs9940099
rs12451892
rs61405042
rs112394943
rs1332941
rs4530278
rs6776756
rs17445108
rs80116508
rs117512405
rs6587577
rs1957937
rs11557154
rs8102497
rs11699829
rs871134
rs76219171
rs2056726
rs869785
rs35446936
rs137901416
rs76065543
rs117630647
rs35640778
rs78491606
rs932002
rs131797
rs7221585
rs7705526
rs9600019
rs80324517
rs144204502
rs2977608
rs13230646
rs73730598
rs6669563
rs1003322
rs73581419
rs11117354
rs9955360
rs3785074
rs1907702
rs2538745
rs185174247
rs6536702
rs611646
rs16978028
rs12412214
rs116863223
rs11991877
rs7790856
rs450962
rs3891167
rs17803849
rs1291143
rs45604339
rs79977579
rs11212631
rs939916
rs202034370
rs56061761
rs41304832
rs10768683
rs66731853
rs10845387
rs2230590
rs150150565
rs111950327
rs11426156
rs76666449
rs10112752
rs1985369
rs141214782
rs6881568
rs11584821
rs11412296
rs9398196
rs4731541
rs117034449
rs28363070
rs188918174
rs11769630
rs34550383
rs10905255
rs6007020
rs10840270
rs376641875
rs117407747
rs4498805
rs113525195
rs61748181
rs2555104
rs4616688
rs7099229
rs12369950
rs139795227
rs10773176
rs111527438
rs9878436
All − MR Egger
All − Inverse variance weighted
−2
−1
0
1
2
MR effect size for
'telomere length || id:ieu−b−4879' on 'IDP_dMRI_TBSS_L3_Superior_longitudinal_fascicu
